# The effect of phosphatidylserine on golf performance

**DOI:** 10.1186/1550-2783-4-23

**Published:** 2007-12-04

**Authors:** Ralf Jäger, Martin Purpura, Kurt-Reiner Geiss, Michael Weiß, Jochen Baumeister, Francesco Amatulli, Lars Schröder, Holger Herwegen

**Affiliations:** 1 Increnovo LLC, 2138 E Lafayette Pl, Milwaukee, WI 53202, USA; 2 ISME, Weingartenstrasse 2, D-64546 Mörfelden-Walldorf, Germany; 3 Department of Sport and Health, University of Paderborn, Warburger Str. 100, 33098 Paderborn, Germany; 4 Department of Sport and Health, Institute of Sports Medicine and Golf Academy, University of Paderborn, Warburger Str. 100, 33098 Paderborn, Germany

## Abstract

**Background:**

A randomized, double-blind, placebo-controlled study was performed to evaluate the effect of oral phosphatidylserine (PS) supplementation on golf performance in healthy young golfers with handicaps of 15–40.

**Methods:**

Perceived stress, heart rate and the quality of the ball flight was evaluated before (pre-test) and after (post-test) 42 days of 200 mg per day PS (n = 10) or placebo (n = 10) intake in the form of a nutritional bar. Subjects teed-off 20 times aiming at a green 135 meters from the tee area.

**Results:**

PS supplementation significantly increased (p < 0.05) the number of good ball flights (mean: pre-test 8.3 ± 3.5, post-test 10.1 ± 3.0), whereas placebo intake (mean: pre-test 7.8 ± 2.4, post-test 7.9 ± 3.6) had no effect. PS supplementation showed a trend towards improving perceived stress levels during teeing-off (mean: pre-test 5.8 ± 2.0, post-test 4.0 ± 2.0, p = 0.07), whereas stress levels remained unchanged in the placebo group (mean: pre-test: 5.1 ± 2.0, post-test: 5.1 ± 3.1). Supplementation did not influence mean heart rate in either group.

**Conclusion:**

It is concluded that six weeks of PS supplementation shows a statistically not significant tendency (p = 0.07) to improve perceived stress levels in golfers and significantly improves (p < 0.05) the number of good ball flights during tee-off which might result in improved golf scores.

## Background

Phosphatidylserine (PS) is an essential component of all biological membranes and is required for normal cellular structure and function. The participation in physical activity often challenges a variety of physiological systems; consequently, the ability to maintain normal cellular function during activity can determine sporting performance. PS has been established as a safe oral supplement [1] capable of attenuating the serum cortisol [2,3] and creatine kinase [4] responses to acute exercise stress. In addition, PS has been reported to improve subjective measures of overtraining such as perceived muscle soreness and well-being [5]. These finding suggest that PS partly counteracts the stress-induced activation of the hypothalamo-pituitary-adrenal (HPA) axis [6].

In addition to physical stress, PS supplementation benefits subjects suffering from mental stress. PS supplementation has been reported to improve mood in a sub-group of healthy young adults when faced with a stressful mental task [7] and blunted both serum ACTH and cortisol, and salivary cortisol responses to the Trier Social Stress Test (TSST) [8].

A recent study by Kingsley et al. showed no effect of oral PS supplementation on markers of muscle damage or perceived soreness, however, PS tended to improve sprint and exercise performances when compared to placebo [9]. These findings do suggest that PS might possess ergogenic properties. The effective daily dosages in sport studies range from 300 to 800 mg PS for short-term application (10–15 days) and from 300 to 400 mg PS for 3 to 4 weeks for mental stress [10]. The lowest effective dose for athletes is yet unknown [10].

These findings suggest that PS supplementation might be beneficial for sports demanding high levels of concentration and coordination such as the game of golf. The golf swing requires the interaction of the central nervous system and skeletal muscles as well as the correct combination of power, velocity and endurance. The golf swing is a complex motion and especially teeing off and putting creates high levels of tension. Salivary cortisol levels have been reported to significantly increase in elite male golfers [11,12]. Individual and external expectations [13] and the importance of the first stroke result in mental stress during tee-off. This might result in negative effects on performance due to an inaccuracy in striking the ball. The tee-off from a stationary location such as a driving range is an ideal study exercise since it allows the easy measure of the effects of stressors on performance parameters such as ball flight. Additional stress can be created by a time restriction between tee offs and by the creation of competitive target task.

Based on this background, the present study assessed the effects of PS supplementation on perceived stress and performance in golfers during tee-off from a driving range. For the first time, a functional food (nutritional bar) was used as study material in a nutritional intervention study on golf performance. This study further investigates the yet lowest daily dose of PS supplementation when faced with a stressful task.

## Methods

### Subjects

Twenty healthy volunteers gave their informed written consent to take part in this study, which was approved by a university ethics committee. The subjects were recruited from the university of Paderborn golf course and other local golf courses. Exclusion criteria on admission were smoking, the use antihistamine or central nervous system medication and apparent physical and mental disease at the time of the study. The subjects were instructed to avoid changes in their diet and their physical, mental and golf specific training habits during the study.

### Study Protocol

**Figure 1:**
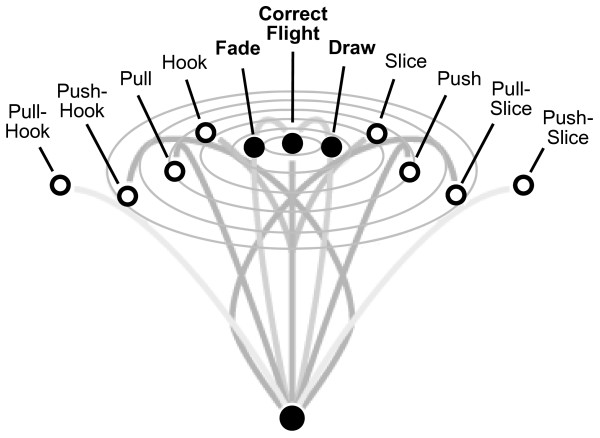
Schematic representation of potential ball flights: correct flight, fade and draw were rated as a hit, all other ball flights were rated as a miss.

A randomized, double-blind, placebo-controlled study was performed over a period of six weeks. Subjects were required to report to the study site on three separate occasions. During pre-screening physical health as well as inclusion (male, handicap 15–40, age 20–55) and exclusion criteria were measured and recorded. The subjects were familiarized with the equipment and the test procedure. Subjects were asked to avoid physical and mental stress, excess food and alcohol intake (limit one beer or one glass of wine) on the day before the pre-test and were instructed to get sufficient night sleep (minimum six hours of sleep). On day one (pre-test) subjects received a standardized breakfast and were not allowed to train or consume caffeine or any other stimulants. Health and overall condition was checked (anamnesis, electrocardiogram, and blood works). Blood works was measured prior to the test to ensure that none of the subjects suffered from a potentially performance reducing disease. The parameters included blood cell count, hemoglobin, glucose, lipids, urea, creatinine, gamma-GT and ferritin. One subject showed hyperferritinemia without signs of a tumor or hemochromatosis and one subject had hyperlipidaemia. No volunteer showed abnormalities that would exclude him from the study. The health check was followed by a standardized warm up which did not include practice shots. The 10 minute warm up consisted of a golf specific eight step warm-up program of one minute each: 1. stationary jumping and running with upper body rotation, 2. upper body rotation and arm lift, 3. back and forth leg weight distribution, 4. upper body rotation simulating swing movement, 5. golf swing with horizontal club, 6. left and right arm one arm swing, 7. left and right arm horizontal swing, 8. golf swing without ball contact, followed by two minutes of golf swings with ball contact. After the warm up subjects teed-off 20 times in 15-second intervals and were asked to hit a target at a distance of 135 meters. The time restriction of 15 seconds was intended to create stress in the study subjects. The hit-a-target task was intended to create competition among subjects and hence to create additional stress. Due to different handicaps subjects were free to choose their individual club (excluding a pitching wedge) which had to be identical for the pre- and post-test. The heart rate was measured using a POLAR S810^® ^heart rate monitor and analyzed with Polar Precision Performance Software (Polar, Finland). Perceived stress was measured after the series of tee-offs using a Visual Analogue Scale (VAS): 1 (low stress) to 10 (maximum stress) [14,15]. The quality of each ball flight was recorded by a professional golf trainer immediately after the ball hit the ground after tee-off. A good ball flight (hit) was defined as "correct flight", "draw" and "fade", whereas all other ball flights were recorded as a miss (see figure 1) [16,17]. The ball flight "draw" starts to the right and ends on target due to a left spin of the ball whereas the ball flight "fade" (starts to the left) hits the target due to a right spin of the ball [16,17].

The subjects reported back to the study side after six weeks (post-test). Subjects reported that their individual physical, mental and golf specific training habits of 2–3 golf training sessions per week were unchanged during the supplementation phase and that they avoided additional physical and mental stress. The individual handicap was identical with pre-study training conditions. Exercise and measurements were repeated under conditions matching the pre-test. The tests were performed in May (pre-test) and June (post-test) during the same time of the day in the morning. The temperature during the tests was measured at 21–23°C. The wind speed was below 1 Beaufort.

### Reliability of the ball flight measurement

The reliability of ball flight expert rating was investigated in a separate test. Two golfers (handicap 0) produced nine good ball flights ("correct flight", "fade" or "draw") and nine misses (all other ball flights) in random order. Two experts rated the ball flights independently from each other. The relationship of two expert ratings was significant (p < 0.001) with a contingency coefficient of C = 0.673. The ball flight measurement is a reliable method to investigate the performance of golfers.

### Experimental conditions

The subjects were assigned in random order to either the PS (n = 10), or the placebo (n = 10) group. The 42-day supplementation period was started immediately after the pre-test and was continued until the day before the post-test. Subjects in the phosphatidylserine group received one nutritional bar (IQ PLUS brain bar, Giventis, Germany) per day containing 200 mg soy-based PS, while the others received corresponding placebo bars. Each bar had a weight of 35 g, providing 149 kcal, 4.8 g protein, 20 g carbohydrates, 5.5 g fat and vitamins (1.4 mg vitamin B1, 1.4 mg vitamin B6, 42 mg vitamin C, 4.6 mg vitamin E, 2.8 niacin and 4.2 mg pantothenic acid).

### Statistics

Statistical analysis was carried out using SPSS software (version 11.0, SPSS Inc., Chicago, IL). Group data were expressed as mean ± SD and statistical significance was set at the p < 0.05 level. Subject characteristics were compared under supplementations groups using independent sample t-tests (table 1). Pre/Post and PS/placebo differences were analyzed by ANOVA (GLM) with post-hoc t-test and Bonferroni correction.

**Table 1:** Subjects characteristics at pre-test

	PS (n = 10)	Placebo (n = 10)
Age (years)	33.1 ± 7.5	31.4 ± 4.5
Bodyweight (kg)	77.6 ± 7.8	84.6 ± 13.2
Height (cm)	181.3 ± 8.8	183.6 ± 5.0
Handicap	26.8 ± 7.5	27.8 ± 8.0

Data are mean ± SD. No significant pre-test group differences were found (p > 0.05).

## Results

### Ball Flight

**Figure 2:**
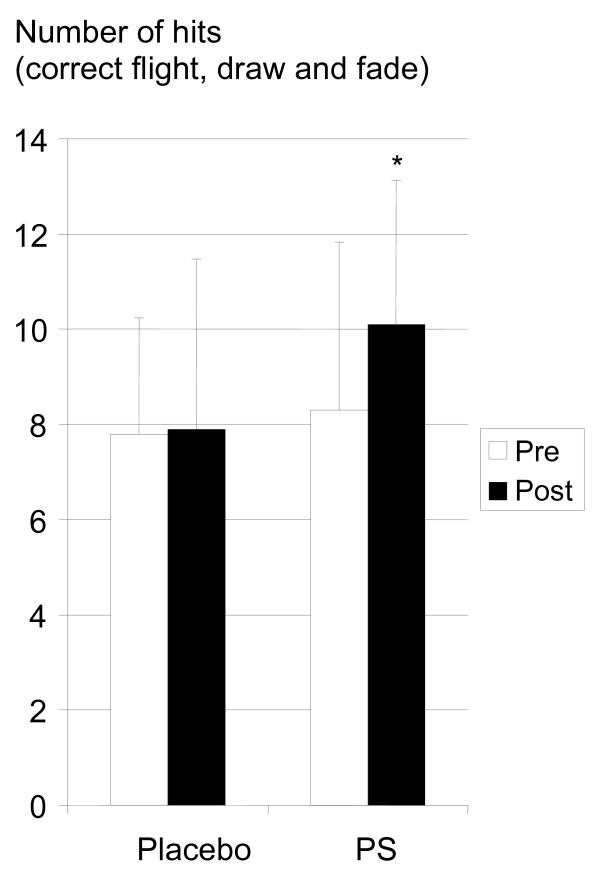
Phosphatidylserine supplementation significantly improves the number of good ball flights.

Phosphatidylserine supplementation significantly increased (<0.05) ball flight accuracy (mean number of good ball flights: pre-test 8.3 ± 3.5, post-test 10.1 ± 3.0), whereas placebo intake (mean number of good ball flights: pre-test 7.8 ± 2.4, post-test 7.9 ± 3.6) had no effect on performance (see figure 2).

### Heart Rate

Phosphatidylserine (mean heart rate: pre-test 113.8 ± 17.7 bpm, post-test 111.3 ± 20.2 bpm) and placebo supplementation (mean heart rate: pre-test 114.3 ± 11.7 bpm, post-test 111.1 ± 15.7 bpm) did not significantly lower mean heart rate during the series of tee-offs.

### Perceived Stress (VAS)

**Figure 3:**
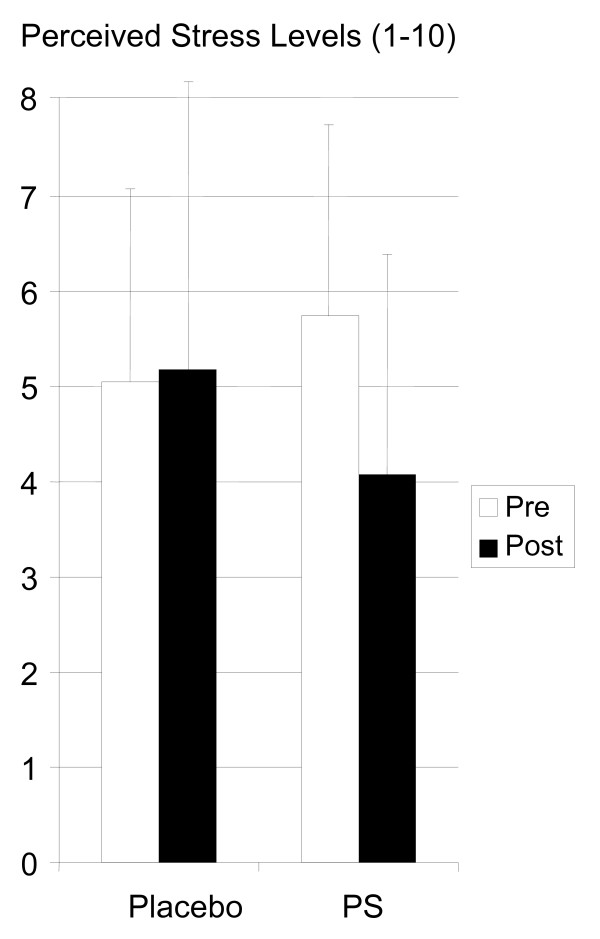
Phosphatidylserine supplementation showed a trend towards improving perceived stress levels during tee-off on a driving range, whereas perceived stress levels remained unchanged in the placebo group.

Phosphatidylserine intake showed a trend towards improving perceived stress levels during tee-off on a driving range (mean: pre-test 5.8 ± 2.0, post-test 4.0 ± 2.0, p = 0.07), whereas perceived stress levels remained unchanged in the placebo group (mean: pre-test: 5.1 ± 2.0, post-test: 5.1 ± 3.1) (see figure 3).

## Discussion

The relationship between stress and sports performance is an extremely complex one and involves the interaction between the nature of the stressor, the cognitive demands of the task being performed and the psychological characteristics of the individuals performing it [18]. Paradoxical performance effects ('choking under pressure') are the result of inferior performance despite striving and incentives for superior performance [19]. Experimental findings of decrease in performance are associated with four pressure variables: audience presence, competition, performance-contingent rewards and punishments, and ego relevance of the task [19].

The primary finding of this investigation was that oral supplementation with 200 mg PS for six weeks in form of a functional bar significantly improved the number of good ball flights in a group of male golfers with a handicap of 15–40. Furthermore, PS intake resulted in a trend of improved perceived stress levels during tee-off. These results suggest that the effective dose of PS supplementation to combat distress might be lower than previously described.

Unpredictability and uncertainty resulting in worry and strain, combined with ego-involvement, are considered the key psychological elements of both HPA activation and distress [8]. While the placebo group showed the expected increase in distress, the PS group showed stable values, suggesting a quicker habituation to the stressor, which may then result in improved performance.

The study protocol did not allow studying the mechanism by which PS affects perceived stress and performance. The very moderate increase in heart rate indicates that physical load did not influence the result of this study. The proposed mechanism of action is the counteraction of stress-induced activation of the hypothalamo-pituitary-adrenal (HPA) axis in accordance with previously reported studies on physical [2,3] and mental stress [8]. However, the proposed mechanism of action remains speculative without supplemental data from further studies that investigate the in vivo pharmacological actions of PS during stress creating golf exercise.

The moderate increase in stress levels in the placebo group indicate that further research is required to investigate the effects of PS supplementation under competition conditions since the elite golfer experienced elevated cortisol, heart rate, cognitive and somatic anxiety, and lower self-confidence during competition compared to practice [13]. The effect of stress on golf performance might be easier to study on subjects with a lower handicap since their golf swing is more automated and more stable under various conditions [11].

## Conclusion

It is concluded that six weeks of PS supplementation shows a statistically not significant tendency (p = 0.07) to improve perceived stress levels in golfers and significantly improves (p < 0.05) the number of good ball flights during tee-off which might result in improved golf scores.

## Competing interests

KRG is the inventor of patent "Food Item For Increasing Cognitive Capacity" (WO 02/078464). All other authors declare that they have no competing interests.

## Authors' contributions

All authors participated in the design of the study. MW analyzed statistics and MW, FA, LS and HH collected data. RJ, with the support of MW and HH, drafted the manuscript. JB validated the ball flight test method. All authors have read and approved the final manuscript.
